# Supplementary material for: p53 rapidly restructures 3D chromatin organization to trigger a transcriptional response
Source: Nat Commun. 2024 Apr 1;15:2821. doi: 10.1038/s41467-024-46666-1 (PMC10984980; doi:10.1038/s41467-024-46666-1)
Supplement: Supplementary file 1 — Supplementary Information [file 41467_2024_46666_MOESM1_ESM.pdf]

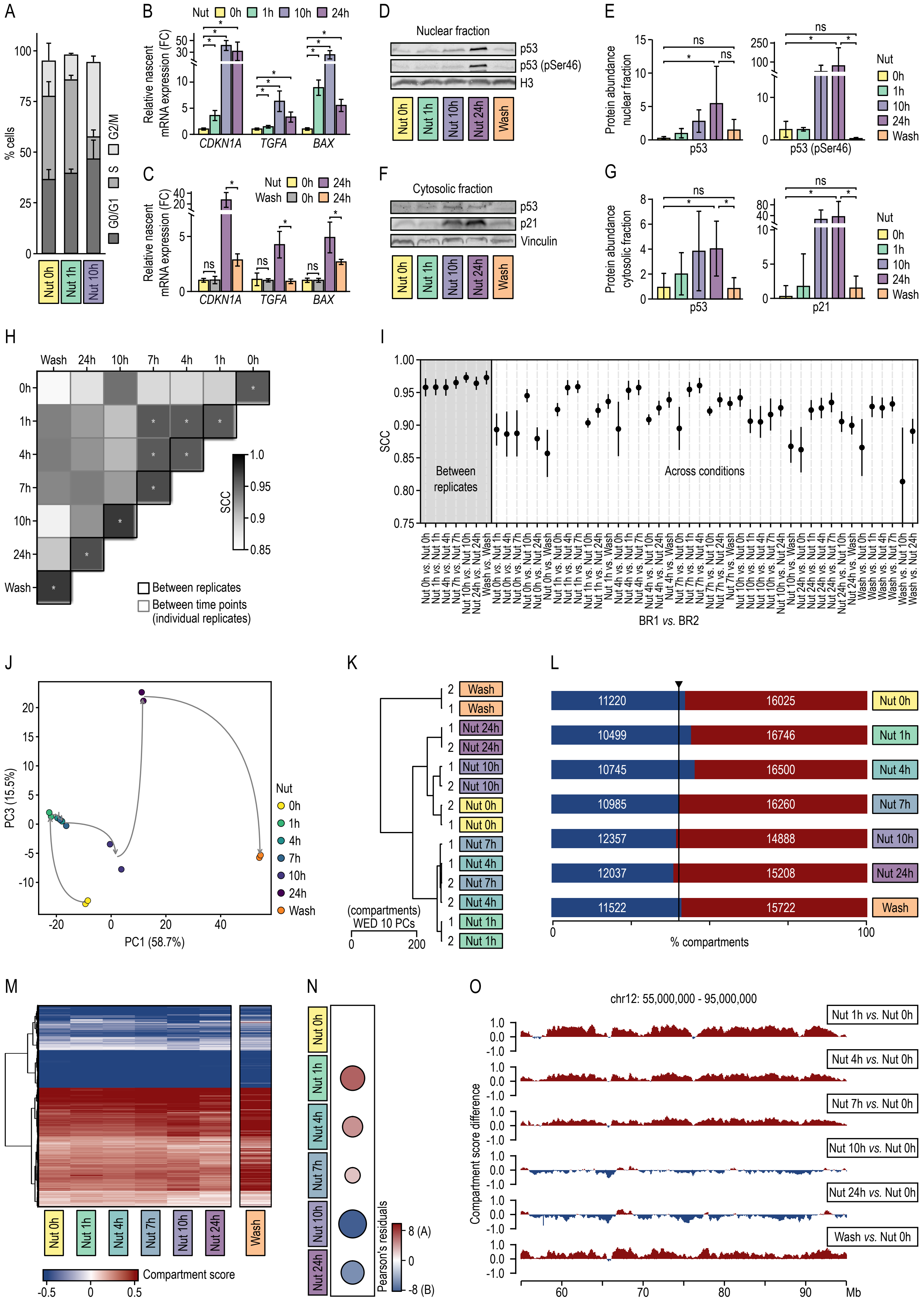
A
% cells
G0/G1
S
G2/M
Nut 0h
Nut 1h
Nut 10h
B
Nut 0h 1h 10h 24h
Relative nascent mRNA expression (FC)
CDKN1A
TGFA
BAX
C
Nut 0h 24h
Wash 0h 24h
ns
D
Nuclear fraction
p53
p53 (pSer46)
H3
Nut 0h
Nut 1h
Nut 10h
Nut 24h
Wash
E
Protein abundance nuclear fraction
p53
p53 (pSer46)
Nut
0h
1h
10h
24h
Wash
F
Cytosolic fraction
p53
p21
Vinculin
G
Protein abundance cytosolic fraction
p53
p21
H
Wash 24h 10h 7h 4h 1h 0h
SCC
Between replicates
Between time points (individual replicates)
I
SCC
Between replicates
Across conditions
BR1 vs. BR2
J
PC3 (15.5%)
PC1 (58.7%)
Nut
0h
1h
4h
7h
10h
24h
Wash
K
(compartments)
WED 10 PCs
Wash
Nut 24h
Nut 10h
Nut 0h
Nut 7h
Nut 4h
Nut 1h
L
11220 16025 Nut 0h
10499 16746 Nut 1h
10745 16500 Nut 4h
10985 16260 Nut 7h
12357 14888 Nut 10h
12037 15208 Nut 24h
11522 15722 Wash
% compartments
M
Nut 0h
Nut 1h
Nut 4h
Nut 7h
Nut 10h
Nut 24h
Wash
Compartment score
N
Pearson's residuals
8 (A)
-8 (B)
O
chr12: 55,000,000 - 95,000,000
Compartment score difference
Nut 1h vs. Nut 0h
Nut 4h vs. Nut 0h
Nut 7h vs. Nut 0h
Nut 10h vs. Nut 0h
Nut 24h vs. Nut 0h
Wash vs. Nut 0h
Mb

**Suppl. Fig. 1 |** Quality controls, reversibility experiments and compartment dynamics

**A.** Distributions of cells in each cell cycle phase (G0–G1, S, G2–M) during Nutlin-3a treatment. Nut 0h means control cells without p53 activation.

**B.** Relative nascent mRNA expression throughout p53 activation for 3 known p53 target genes, measured by qRT-PCR.

**C.** Relative nascent mRNA expression 0 and 24 hours after p53 activation, before (Nut) and after (Wash) washing Nutlin-3a.

**D.** Western blot of nuclear fraction of p53 and activated p53 as marked by the phosphorylation of serine 46 (p53 (pSer46)) at 0, 1, 10, and 24 hour timepoints and after washing Nutlin-3a, using Histone 3 (H3) as control. Source data is provided in Source Data file.

**E.** Bar plot showing quantification of western blot show in **D**.

**F.** Western blot of cytosolic fraction of p53 and p21 (CDKN1A) at 0, 1, 10, and 24 timepoints and after washing Nutlin-3a, using Vinculin as control. Source data is provided in Source Data file.

**G.** Bar plot showing quantification of western blot shown in **F**.

**H.** Similarity between Hi-C interaction matrices calculated using the Stratum Adjusted Correlation Coefficient (SCC) throughout p53 activation. SCC of 1 means perfect correlation, values above 0.95 (star) are expected for biological replicates.

**I.** Stratum Adjusted Correlation Coefficient (SCC), with standard deviation bar, between time points and replicates of Hi-C data.

**J.** Principal Component Analysis of compartment scores for all Hi-C biological replicates throughout p53 activation and inactivation. Principal components (PC) 1 and 3 are shown. Numbers in parentheses represent the percentage of variance explained by each PC.

**K.** Hierarchical clustering (Ward criterion) of compartment values throughout p53 activation and inactivation. Distances between replicates were measured by applying weighted Euclidean distance (WED) of the 10 first PCs.

**L.** Percentage of 100kb genomic regions defined as A or B compartments for each timepoint.

**M.** Compartment scores of the non-dynamic compartments upon p53 activation and inactivation. Each row represents a 100kb bin and each column represents a time point.

**N.** Correlation plot of Pearson's residuals from a Chi-square test of independence for the contingency table time-course *vs.* compartment category (X-squared = 410.52, df = 5, p-value < 2.2e-16). Only significant associations (with |Pearson's residual| > 2) are shown. Bubble size indicates the number of compartment regions associated.

**O.** Difference in compartment score between p53 activated and non-activated cells (Nut 0h) along the region of chromosome 12 displayed in **Figure 1E**. Positive values in red indicate a shift towards the A compartment throughout p53 activation, while negative values in blue indicate a shift towards the B compartment.

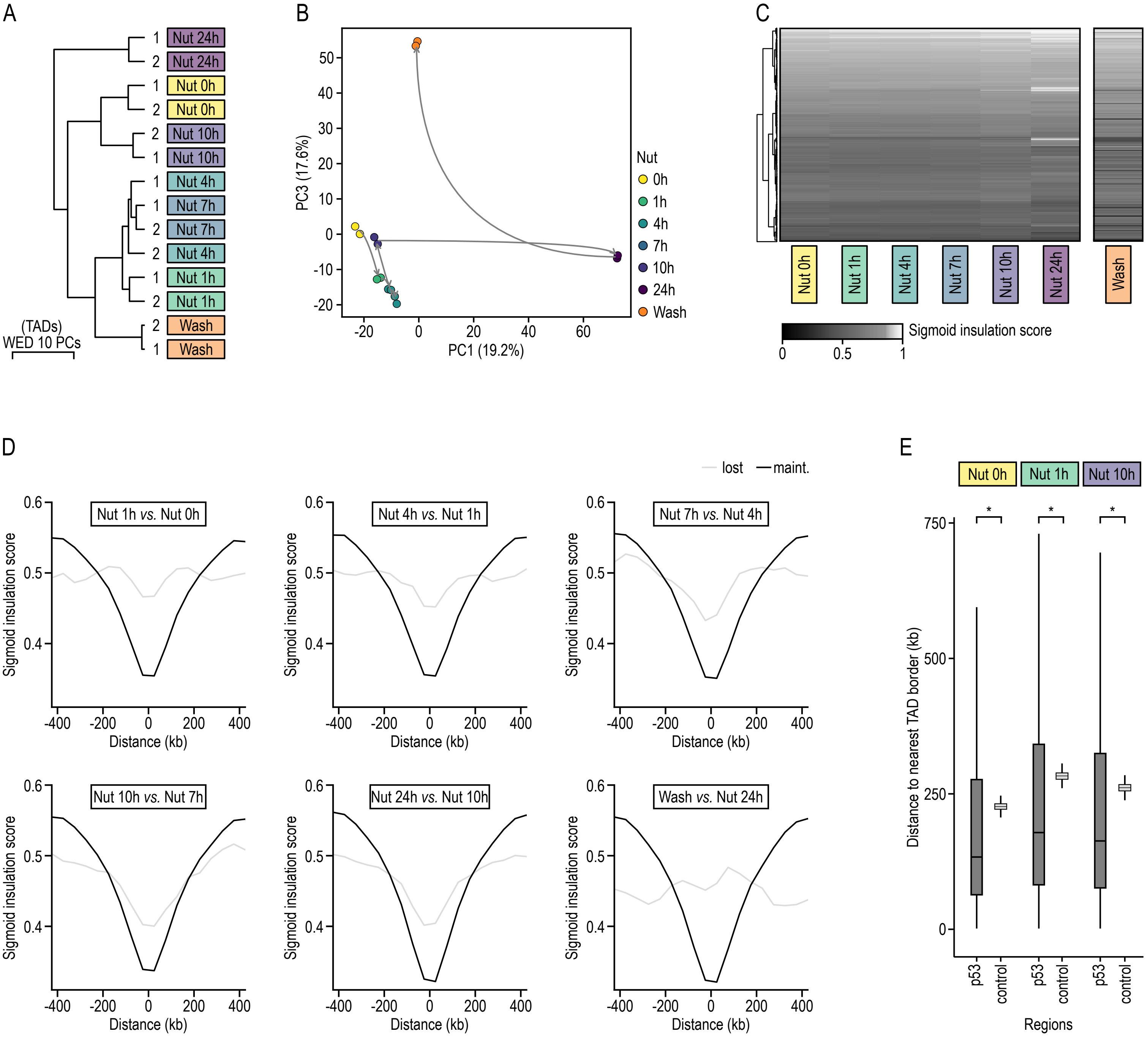
A
Nut 24h
Nut 0h
Nut 10h
Nut 4h
Nut 7h
Nut 1h
Wash
(TADs)
WED 10 PCs
B
PC3 (17.6%)
PC1 (19.2%)
Nut
0h
1h
4h
7h
10h
24h
Wash
C
Sigmoid insulation score
D
lost
maint.
Nut 1h vs. Nut 0h
Nut 4h vs. Nut 1h
Nut 7h vs. Nut 4h
Nut 10h vs. Nut 7h
Nut 24h vs. Nut 10h
Wash vs. Nut 24h
Sigmoid insulation score
Distance (kb)
E
Nut 0h
Nut 1h
Nut 10h
Distance to nearest TAD border (kb)
p53
control
Regions

**Suppl. Fig. 2 |** Topologically associated domain dynamics

**A.** Hierarchical clustering analysis (Ward criterion) of TADbit TAD border scores across p53 activation and after washing of Nutlin-3a, reflecting the degree of dissimilarity between biological replicates of different samples. The distances between replicates were measured by applying weighted Euclidean distance (WED) of the 10 first principal components.

**B.** Principal Component Analysis of TADbit TAD border scores for all Hi-C biological replicates throughout p53 activation and after washing Nutlin-3a. Principal components 1 and 3 are shown. Numbers in parentheses represent the percentage of variance explained by each principal component (PC).

**C.** Sigmoid-normalized TAD insulation scores of the TAD borders that remained unchanged across all samples (invariant TAD borders). Colour intensity represents insulation score, with lower scores (in black) indicating stronger insulation between TADs.

**D.** Insulation score profiles stacked over a 1Mb genomic region centred at TAD borders. Only TAD borders characterized by a TADbit score > 4 and identified as invariant (maint.) or lost across adjacent time points were included. Sigmoid insulation score is inversely proportional to insulation capacity.

**E.** Distribution of the distances between functional "p53" binding sites, or "control" regions, and the nearest TAD border. Control regions are defined as a random set of regions of similar genomic length to a p53 binding site and a similar distribution across the genome. Statistical comparisons between p53 and control regions were computed using a two-sided Wilcoxon test.

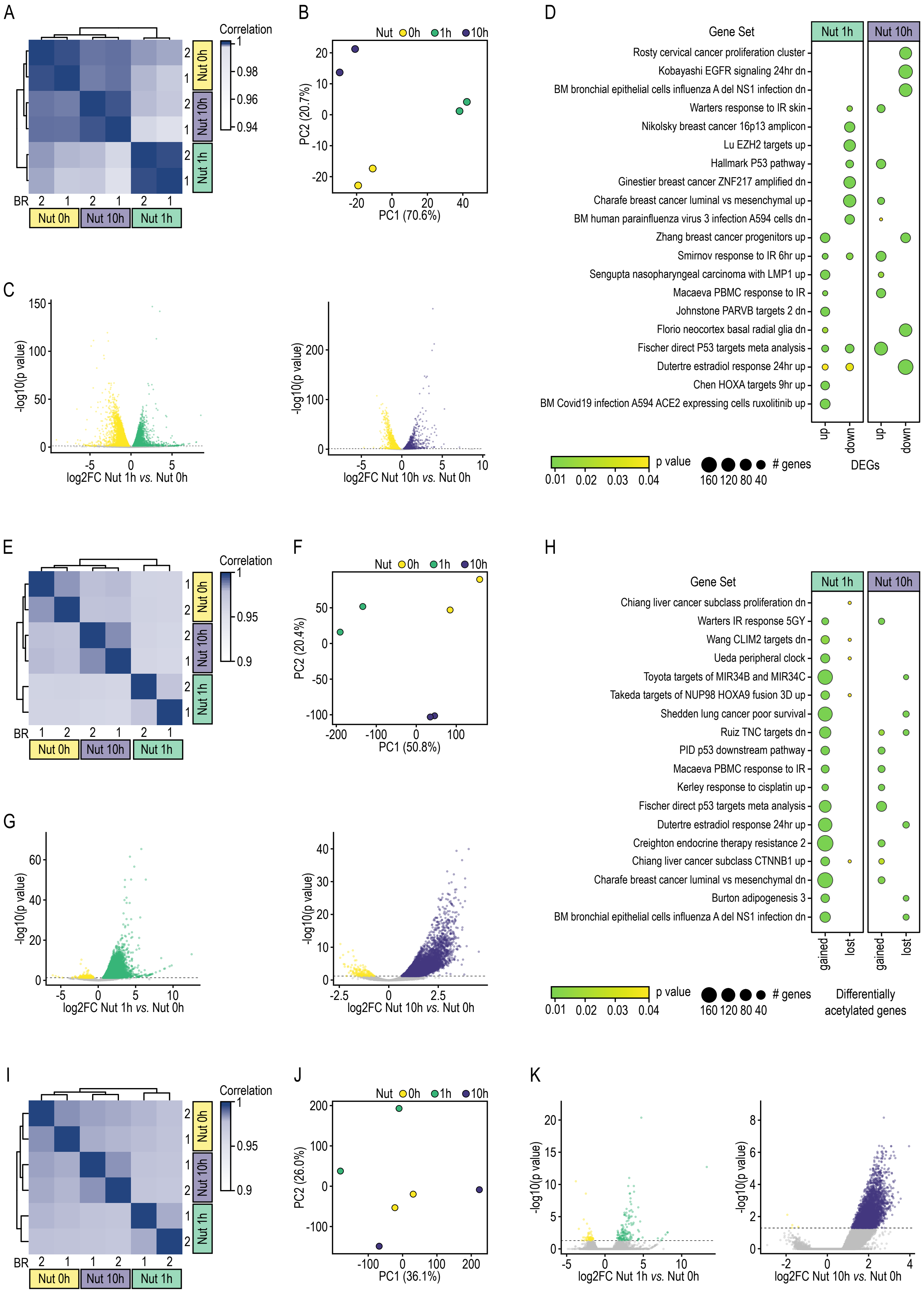
A
Correlation
1
0.98
0.96
0.94
BR
Nut 0h
Nut 10h
Nut 1h
B
Nut 0h 1h 10h
PC2 (20.7%)
PC1 (70.6%)
C
-log10(p value)
log2FC Nut 1h vs. Nut 0h
log2FC Nut 10h vs. Nut 0h
D
Gene Set
Nut 1h
Nut 10h
Rosty cervical cancer proliferation cluster
Kobayashi EGFR signaling 24hr dn
BM bronchial epithelial cells influenza A del NS1 infection dn
Warters response to IR skin
Nikolsky breast cancer 16p13 amplicon
Lu EZH2 targets up
Hallmark P53 pathway
Ginestier breast cancer ZNF217 amplified dn
Charafe breast cancer luminal vs mesenchymal up
BM human parainfluenza virus 3 infection A594 cells dn
Zhang breast cancer progenitors up
Smirnov response to IR 6hr up
Sengupta nasopharyngeal carcinoma with LMP1 up
Macaeva PBMC response to IR
Johnstone PARVB targets 2 dn
Florio neocortex basal radial glia dn
Fischer direct P53 targets meta analysis
Dutertre estradiol response 24hr up
Chen HOXA targets 9hr up
BM Covid19 infection A594 ACE2 expressing cells ruxolitinib up
up
down
p value
0.01 0.02 0.03 0.04
# genes
160 120 80 40
DEGs
E
Correlation
1
0.95
0.9
BR
Nut 0h
Nut 10h
Nut 1h
F
Nut 0h 1h 10h
PC2 (20.4%)
PC1 (50.8%)
G
-log10(p value)
log2FC Nut 1h vs. Nut 0h
log2FC Nut 10h vs. Nut 0h
H
Gene Set
Nut 1h
Nut 10h
Chiang liver cancer subclass proliferation dn
Warters IR response 5GY
Wang CLIM2 targets dn
Ueda peripheral clock
Toyota targets of MIR34B and MIR34C
Takeda targets of NUP98 HOXA9 fusion 3D up
Shedden lung cancer poor survival
Ruiz TNC targets dn
PID p53 downstream pathway
Macaeva PBMC response to IR
Kerley response to cisplatin up
Fischer direct p53 targets meta analysis
Dutertre estradiol response 24hr up
Creighton endocrine therapy resistance 2
Chiang liver cancer subclass CTNNB1 up
Charafe breast cancer luminal vs mesenchymal dn
Burton adipogenesis 3
BM bronchial epithelial cells influenza A del NS1 infection dn
gained
lost
p value
0.01 0.02 0.03 0.04
# genes
160 120 80 40
Differentially
acetylated genes
I
Correlation
1
0.95
0.9
BR
Nut 0h
Nut 10h
Nut 1h
J
Nut 0h 1h 10h
PC2 (26.0%)
PC1 (36.1%)
K
-log10(p value)
log2FC Nut 1h vs. Nut 0h
log2FC Nut 10h vs. Nut 0h

**Suppl. Fig. 3 |** Pre-processing of RNA-seq and ChIP-seq datasets

**A.** Pearson Correlation between biological replicates (1 and 2) of RNA-seq samples along p53 activation. Samples were clustered hierarchically inputting Euclidean distances to the "complete" agglomeration method. Nut 0h refers to control cells without p53 activation.

**B.** Principal Component Analysis of RNA-seq biological replicates along p53 activation. Numbers in parentheses represent the percentage of variance explained by each principal component (PC).

**C.** Differential expression analysis between 1 hour (Nut 1h) and 0 hour (Nut 0h) time points (left) and 10 hour (Nut 10h) and 0 hour (Nut 0h) time points (right). Differentially expressed genes are those with log2 fold change (log2FC) $\neq 0$ and adjusted P value $\leq 0.05$ (horizontal dotted line). Upregulated genes are in green or purple, downregulated genes are in yellow.

**D.** Gene sets from the Molecular Signature Database (MSigDB) with significant enrichment (p-adj $< 0.05$) in one or more gene groups defined using differentially expressed genes (DEGS; upregulated as up; downregulated as down) comparing Nut 0h to Nut 1h and Nut 10h time points. Bubble size indicates the number of genes found in a gene set. P-values were calculated using a hypergeometric distribution test and adjusted for multiple comparison using a Benjamini-Hochberg (BH).

**E, F, G** and **H.** As panel A, B, C and D respectively, but for H3K27ac ChIP-seq data.

**I, J** and **K.** As panel A, B and C respectively but for H3K4me1 ChIP-seq data.

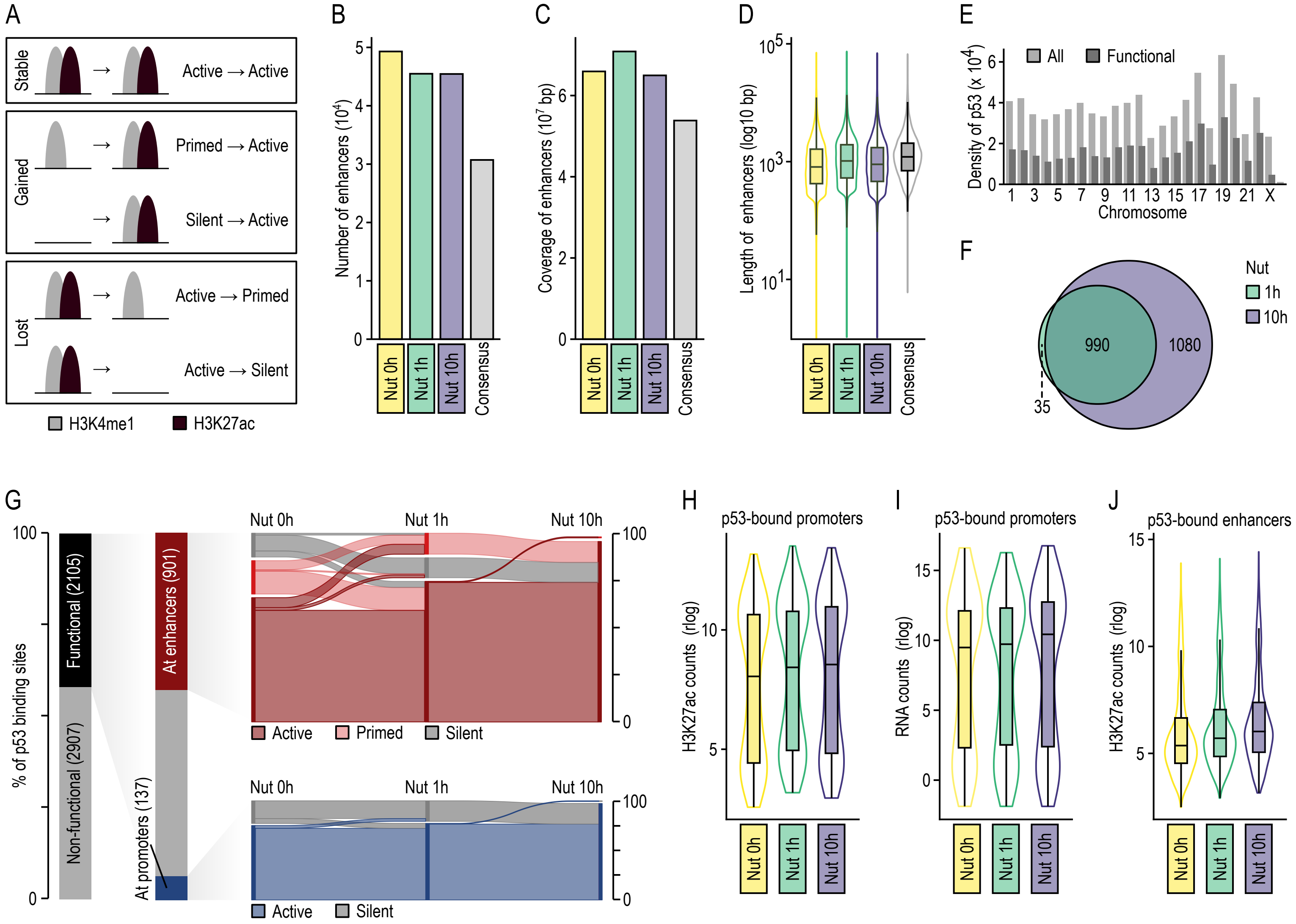

A
Stable
Active → Active
Gained
Primed → Active
Silent → Active
Lost
Active → Primed
Active → Silent
H3K4me1
H3K27ac
B
Number of enhancers (10⁴)
Nut 0h
Nut 1h
Nut 10h
Consensus
C
Coverage of enhancers (10⁷ bp)
D
Length of enhancers (log10 bp)
E
All
Functional
Density of p53 (x 10⁴)
Chromosome
F
Nut
1h
10h
990
1080
35
G
% of p53 binding sites
Functional (2105)
Non-functional (2907)
At enhancers (901)
At promoters (137)
Nut 0h
Nut 1h
Nut 10h
Active
Primed
Silent
H
p53-bound promoters
H3K27ac counts (rlog)
I
p53-bound promoters
RNA counts (rlog)
J
p53-bound enhancers
H3K27ac counts (rlog)

**Suppl. Fig. 4 |** Characterizing functional p53 binding sites and enhancers

**A.** Schematic representation of our definition of enhancer dynamism.
**B.** Number of enhancers defined at time points Nut 0h, Nut 1h and Nut 10h, as well as the consensus number of enhancers reached by intersecting the data of all three time points. Enhancers are defined as regions of the genome covered by H3K27ac and H3K4me1 peaks. Nut 0h refers to control cells without p53 activation.
**C.** Genomic coverage of the enhancers defined at time points Nut 0h, Nut 1h and Nut 10h, as well as for the consensus set enhancers.
**D.** Distribution of enhancer sizes defined at time points Nut 0h, Nut 1h and Nut 10h, as well as in the consensus set of enhancers.
**E.** Number of all (light grey) and functional (dark grey) p53 binding sites per chromosome. Functional p53 binding sites are defined as p53 binding sites with an overlapping H3K27ac peak at either Nut 1h or Nut 10h time points.
**F.** Overlap between functional p53 binding sites defined at Nut 1h and Nut 10h time points.
**G.** Descriptive representation of the defined proportion of functional p53 binding sites (black) found at either promoters (blue) or enhancers (red) with varying states of activity over defined time points (Nut 0h, Nut 1h and Nut 10h).
**H.** Distribution of normalized and rlog transformed H3K27ac counts at active promoter elements over the three time points (Nut 0h, Nut 1h and Nut 10h).
**I.** Distribution of normalized and rlog transformed RNA-seq counts of genes with active promoter elements along p53 activation.
**J.** Distribution of normalized and rlog transformed H3K27ac counts at active enhancers over the three time points (Nut 0h, Nut 1h and Nut 10h).

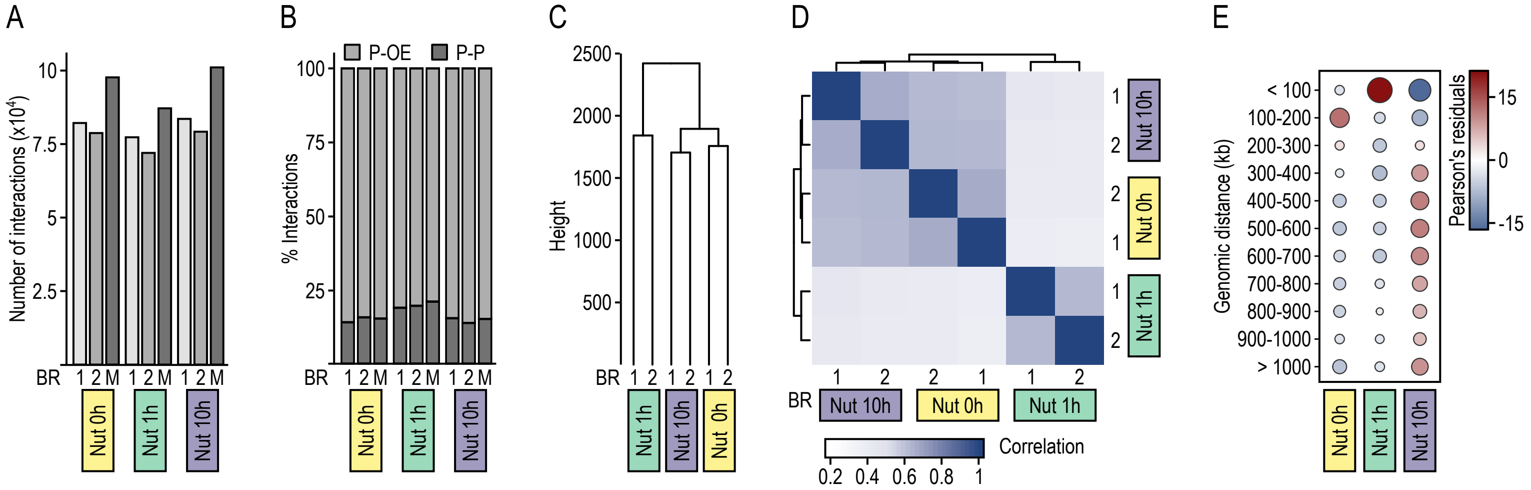
A
Number of interactions (x10^4)
10
7.5
5
2.5
BR
1 2 M 1 2 M 1 2 M
Nut 0h
Nut 1h
Nut 10h
B
P-OE
P-P
% Interactions
100
75
50
25
C
Height
2500
2000
1500
1000
500
D
Correlation
0.2 0.4 0.6 0.8 1
E
Genomic distance (kb)
< 100
100-200
200-300
300-400
400-500
500-600
600-700
700-800
800-900
900-1000
> 1000
Pearson's residuals
15
0
-15

**Suppl. Fig. 5 |** Pre-processing of PCHi-C datasets

**A.** Total number of significant promoter interactions (CHICAGO score ≥ 5) of biological replicates (1 and 2) and merged samples (M) obtained by PCHi-C at Nut 0h, Nut 1h and Nut 10h time points. Nut 0h refers to control cells without p53 activation.

**B.** Percentage of promoter-promoter (P-P) and promoter-other end (P-OE) significant interactions of biological replicates (1 and 2) and merged samples (M) obtained by PCHi-C along p53 activation.

**C.** Hierarchical clustering of biological replicates (1 and 2) of promoter interactomes obtained by PCHi-C at Nut 0h, Nut 1h and Nut 10h time points. Euclidean distances were measured between samples, and the "complete" agglomeration method was used.

**D.** Pearson correlations between promoter interactomes obtained by PCHi-C of biological replicates at 0, 1 and 10 hour time points. Samples were clustered hierarchically using Euclidean distances and the "complete" agglomeration method. Nut 0h refers to control cells without p53 activation.

**E.** Correlation plot for resultant Pearson's residuals from a Chi-square test of independence for the contingency table time-course *vs.* genomic distance bracket (X-squared = 2096.3, df = 20, p-value < 2.2e-16). Only significant associations (with | Pearson's residual| > 2) are shown. Bubble size indicates the number of interactions associated. Blue to red corresponds to an increasing association with genomic distance bracket.

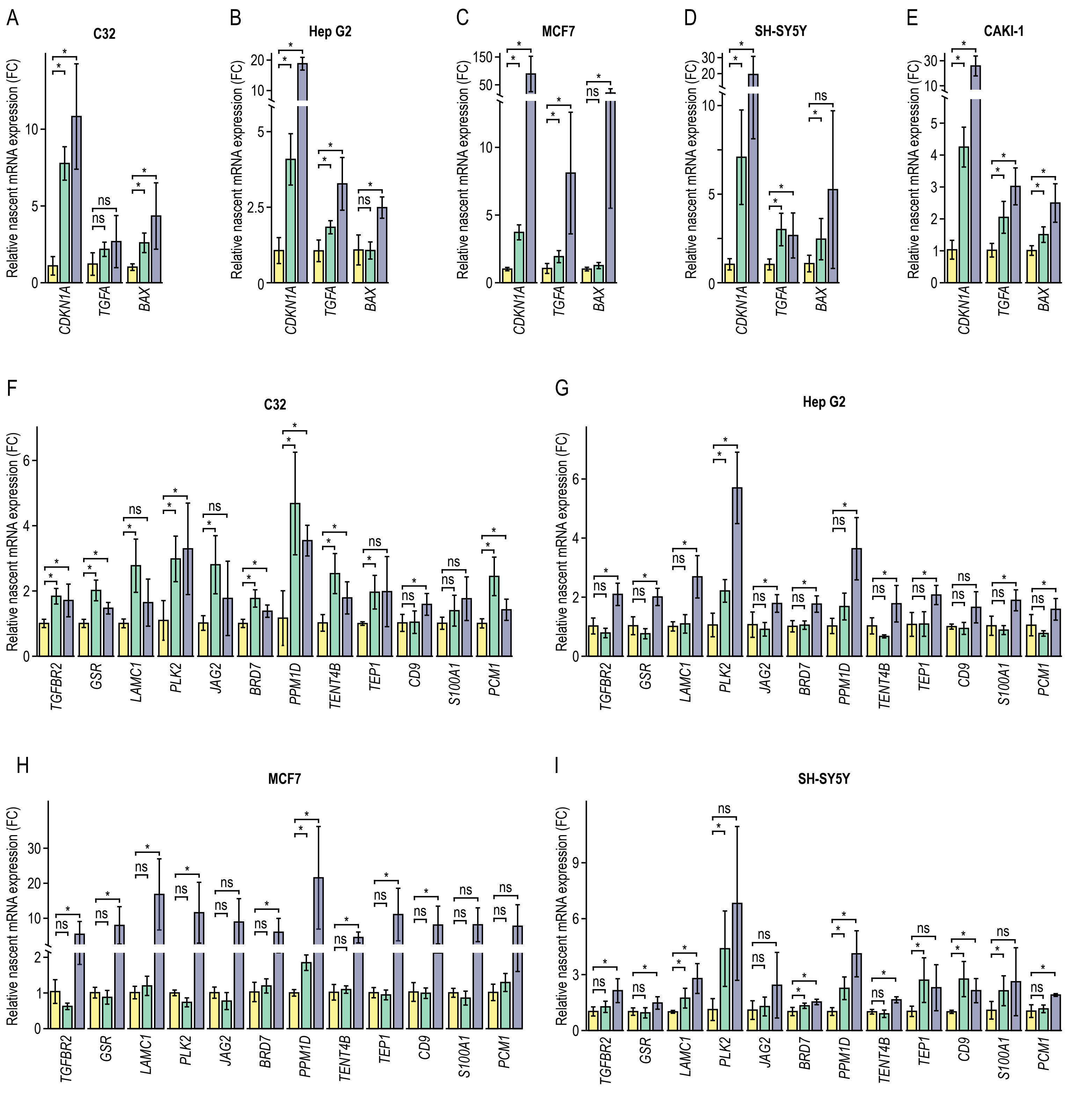


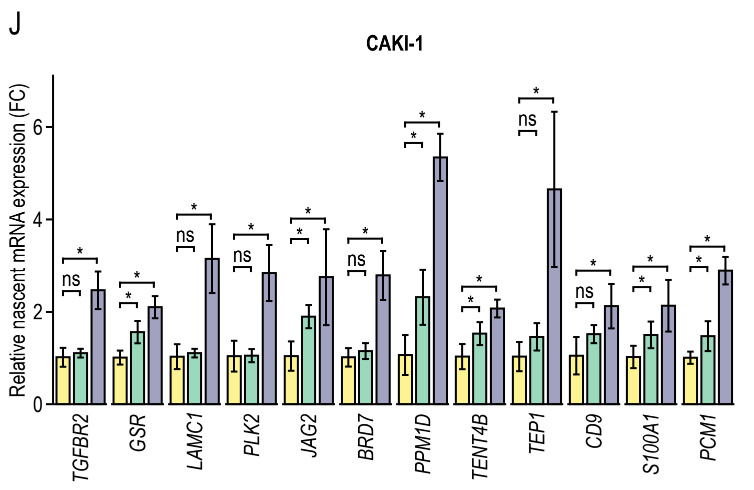


K

| Cell line | Cancer type | Tissue |
|---|---|---|
| C32 | Amelanotic Melanoma | Skin |
| Hep G2 | Hepatocellular Carcinoma | Liver |
| MCF7 | Adenocarcinoma | Mammary gland |
| SH-SY5Y | Neuroblastoma | Bone Marrow |
| CAKI-1 | Clear cell Carcinoma | Kidney |

**Suppl. Fig. 6 |** Testing p53 distal target genes in a five different cell lines

**A.** Levels of relative nascent mRNA expression along p53 activation for 3 known p53 target genes in C32 cell line, measured by qRT-PCR.

**B, C, D** and **E.** As in panel A but for HepG2, MCF7, SH-SY5Y and CAKI-1 cell lines, respectively.

**F.** Relative nascent mRNA expression (fold change) of p53 distal target genes along p53 activation in C32 cell line, measured by qRT-PCR. A one-tailed Student's t-test was used to test whether relative expression differed significantly between adjacent time points for each gene (star).

**G, H, I,** and **J.** As in panel F for HepG2, MCF7, SH-SY5Y and CAKI-1 cell lines, respectively.

**K.** Table summarizing information on additional cell lines used.

A

| Cell type | Acronym | Developmental origin |
|---|---|---|
| Primary Human Dermal Lymphatic Endothelial Cells | HDLECs | Mesoderm |
| Primary Human Umbilical Vein Endothelial Cells | HUVECs | Mesoderm |
| Pericytes | - | Mesoderm |

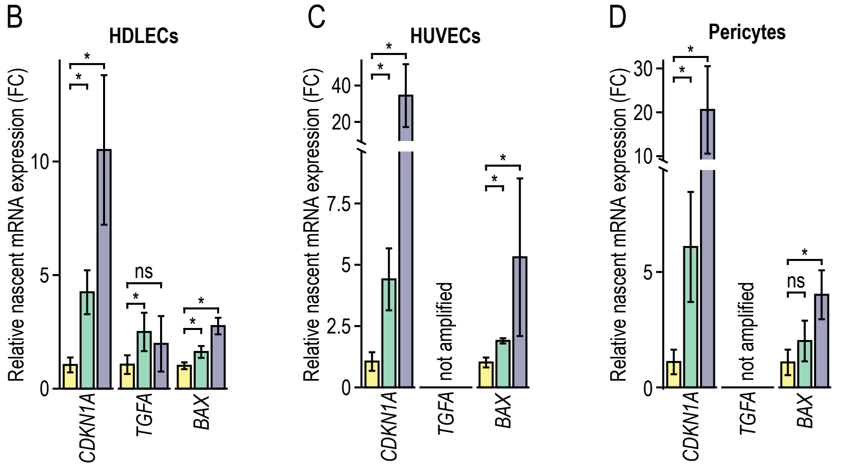


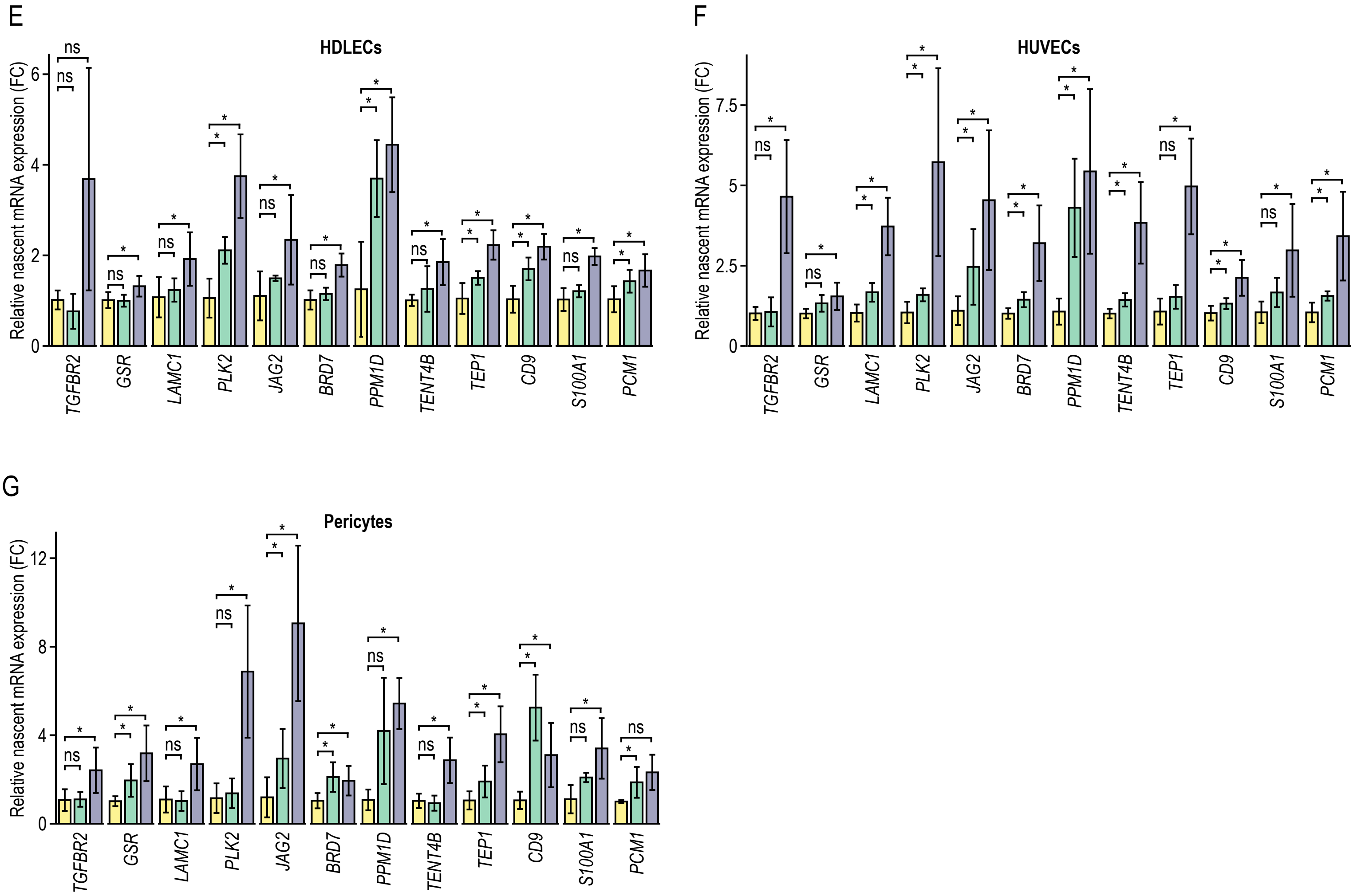

**Suppl. Fig. 7 |** Testing p53 distal target genes in three different primary cell types

**A.** Table summarizing information on additional cell types used.
**B.** Levels of relative nascent mRNA expression along p53 activation for 3 known p53 target genes in primary human dermal lymphatic endothelial cells (HDLECs), measured by qRT-PCR.
**C** and **D.** As in panel B but for primary human umbilical vein endothelial cells (HUVECs) and Pericytes, respectively.
**E.** Relative nascent mRNA expression (fold change) of p53 distal target genes along p53 activation in HDLECs, measured by qRT-PCR. A one-tailed Student's t-test was used to test whether relative expression differed significantly between adjacent time points for each gene (star).
**F,** and **G.** As in panel E but for HUVECs and Pericytes, respectively.

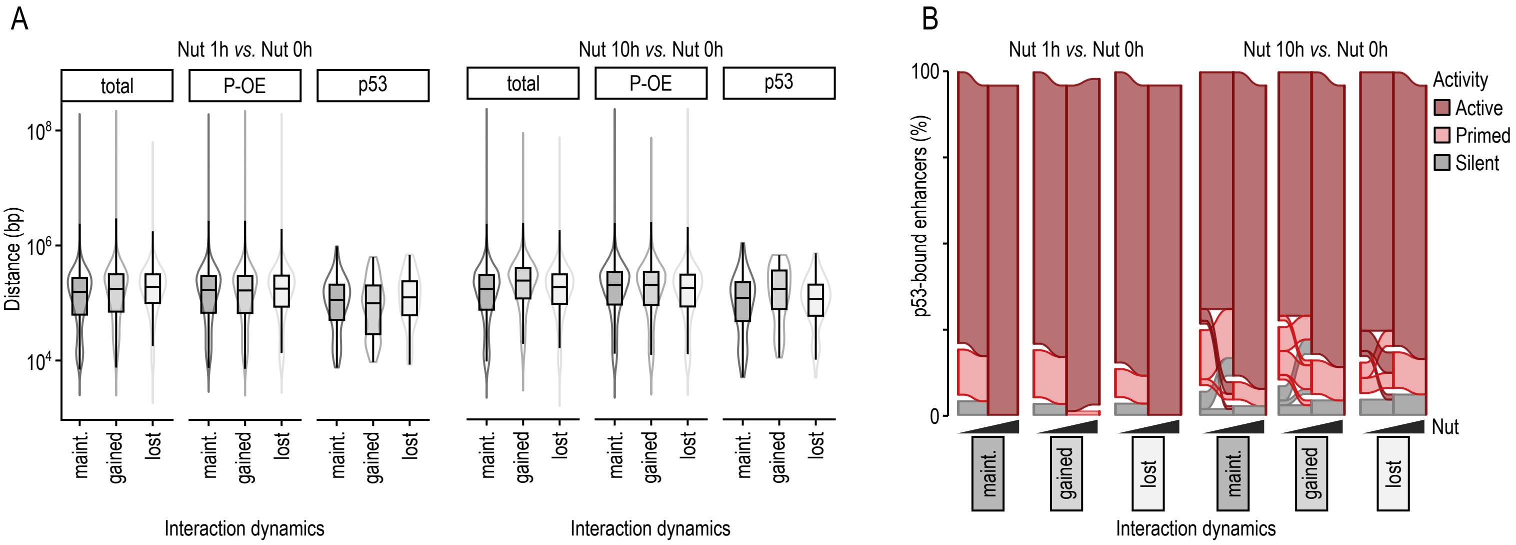

A
Nut 1h vs. Nut 0h
Nut 10h vs. Nut 0h
total
P-OE
p53
Distance (bp)
maint.
gained
lost
Interaction dynamics
B
p53-bound enhancers (%)
Activity
Active
Primed
Silent
Nut

**Suppl. Fig. 8 |** Characterizing interaction dynamics between p53-bound enhancers and distal target genes

**A.** Distribution of distances of significant promoter interactions (CHiCAGO score ≥ 5) maintained, gained and lost between Nut 1h and Nut 0h time points (left) and Nut 10h and Nut 0h time points (right) respectively. The three interaction sets shown are comparing the complete interactomes (total); comparing interactions between a promoter and a non-promoter region (promoter with other-end or P-OE); and comparing interactomes with a p53 binding site present at either end (p53). Nut 0h refers to control cells prior to p53 activation.

**B.** Sankey plot showing the activity dynamics of p53-bound enhancers after 1 hour (left) and 10 hours of p53 activation (right). Trends are divided considering only interactions maintained, gained or lost.

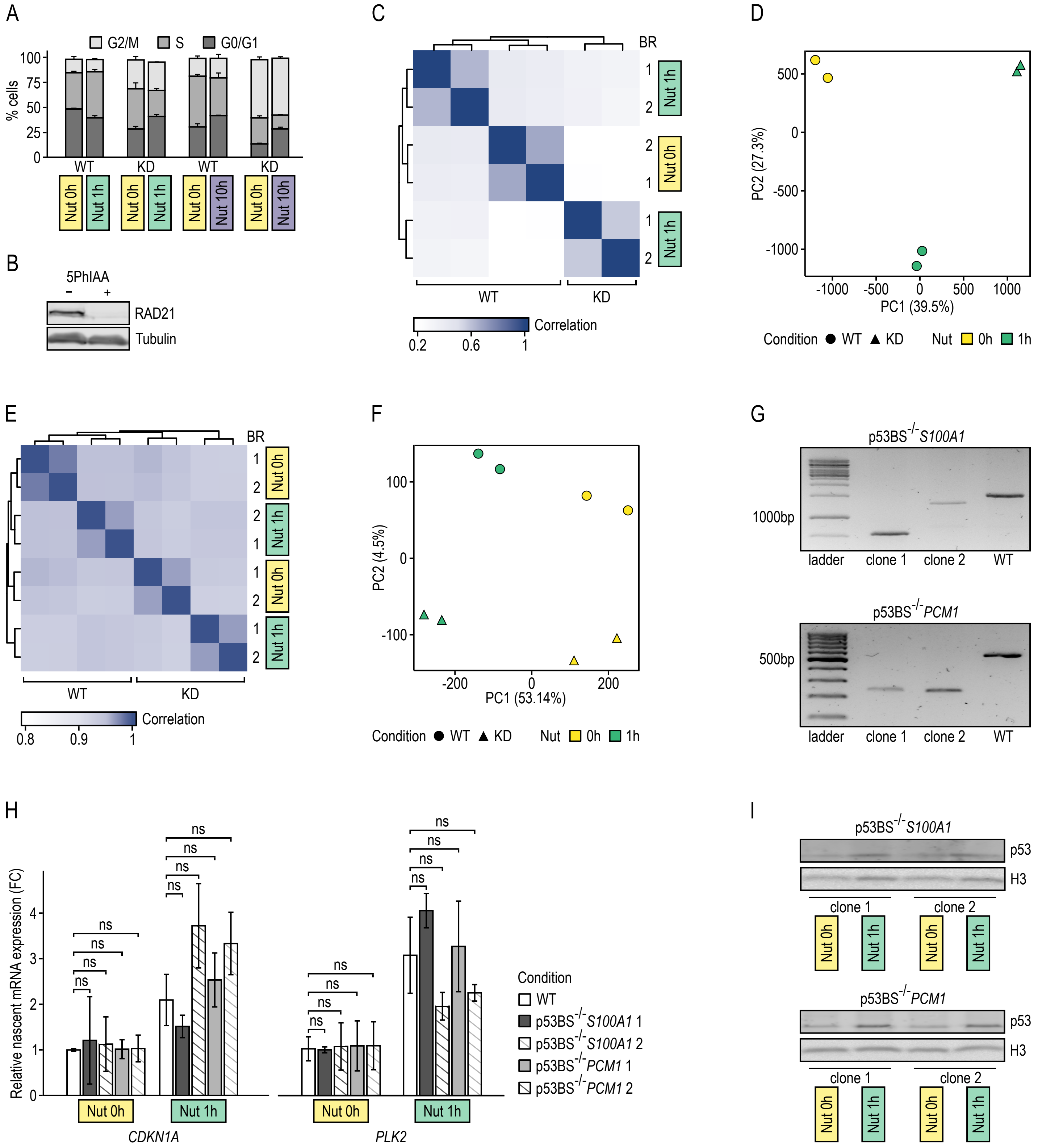

A
G2/M S G0/G1
% cells
WT KD WT KD
Nut 0h Nut 1h Nut 0h Nut 1h Nut 0h Nut 10h Nut 0h Nut 10h
B
5PhIAA
RAD21
Tubulin
C
BR
Correlation
WT KD
Nut 1h Nut 0h Nut 1h
D
PC2 (27.3%)
PC1 (39.5%)
Condition WT KD Nut 0h 1h
E
BR
Correlation
WT KD
Nut 0h Nut 1h Nut 0h Nut 1h
F
PC2 (4.5%)
PC1 (53.14%)
Condition WT KD Nut 0h 1h
G
p53BS-/- S100A1
1000bp
ladder clone 1 clone 2 WT
p53BS-/- PCM1
500bp
ladder clone 1 clone 2 WT
H
Relative nascent mRNA expression (FC)
ns
Nut 0h Nut 1h
CDKN1A
PLK2
Condition
WT
p53BS-/- S100A1 1
p53BS-/- S100A1 2
p53BS-/- PCM1 1
p53BS-/- PCM1 2
I
p53BS-/- S100A1
p53
H3
clone 1 clone 2
Nut 0h Nut 1h Nut 0h Nut 1h
p53BS-/- PCM1
p53
H3
clone 1 clone 2
Nut 0h Nut 1h Nut 0h Nut 1h

**Suppl. Fig. 9 |** Functional validation of p53 distal target genes

**A.** Representative distributions of cells in each cell cycle phase (G0–G1, S, G2–M) during Nutlin-3a treatment in the presence (WT) or absence of RAD21 (RAD21 KD).
**B.** Western blot of RAD21 at with and without auxin treatment (*i.e.,* 5PhIAA), using Tubulin as control. Source data is provided in Source Data file.
**C.** Pearson correlation between biological replicates (1 and 2) of PCHi-C samples at time points Nut 0h and Nut 1h, both in wild type (WT) and knockdown condition of RAD21 (KD) using an auxin inducible degron system. Samples were clustered hierarchically using Euclidean distances and the "complete" agglomeration method. Nut 0h refers to control cells prior to p53 activation.
**D.** Principal Component Analysis of PCHi-C biological replicates of time point Nut 0h and Nut 1h both in wild type (WT) and knockdown condition of RAD21 (KD). Numbers in parentheses represent the percentage of variance explained by each principal component (PC).
**E.** Pearson correlation between biological replicates (1 and 2) of H3K27ac ChIP-seq samples at time points Nut 0h and Nut 1h, both in wild type (WT) and knockdown condition of RAD21 (KD) using an auxin inducible degron system. Samples were clustered hierarchically using Euclidean distances and the "complete" agglomeration method. Nut 0h refers to control cells prior to p53 activation.
**F.** Principal Component Analysis of H3K27ac ChIP-seq biological replicates of time point Nut 0h and Nut 1h both in wild type (WT) and knockdown condition of RAD21 (KD). Numbers in parentheses represent the percentage of variance explained by each principal component (PC).
**G.** PCR amplification of p53 binding sites targeting distal target genes *S100A1* and *PCM1* (top: p53BS$^{-/-}$ *S100A1*, bottom: p53BS$^{-/-}$ *PCM1*) in CRISPR deletion clones 1 and 2 and wild type (WT) cells.
**H.** Levels of relative nascent mRNA expression at 0 and 1 hour after p53 activation for well-known p53 target gene *CDKN1A* and characterized distal target genes *PLK2* in wild type (WT) and CRISPR clones (p53BS$^{-/-}$ *S100A1,* p53BS$^{-/-}$ *PCM1*), measured by qRT-PCR.
**I.** Western blot of p53 for both CRISPR clones (p53BS$^{-/-}$ *S100A1,* p53BS$^{-/-}$ *PCM1*) targeting distal p53 binding sites of top: *S100A1* and bottom: *PCM1*, using Histone 3 (H3) as control. Source data is provided in Source Data file.
