# Supplementary material for: p53 rapidly restructures 3D chromatin organization to trigger a transcriptional response
Source: Nat Commun. 2024 Apr 1;15:2821. doi: 10.1038/s41467-024-46666-1 (PMC10984980; doi:10.1038/s41467-024-46666-1)
Supplement: Supplementary file 3 — Description of Additional Supplementary Files [file 41467_2024_46666_MOESM3_ESM.pdf]

Description of Additional Supplementary Files

File Name: Supplementary Data 1
Description: Western blot quantifications

File Name: Supplementary Data 2
Description: Relative nascent expression values obtained by qRT-PCR

File Name: Supplementary Data 3
Description: Pre-processing statistics of Hi-C datasets.

File Name: Supplementary Data 4
Description: Pre-processing statistics of ChIP-seq datasets.

File Name: Supplementary Data 5
Description: Pre-processing statistics of RNA-seq datasets.

File Name: Supplementary Data 6
Description: Characterization of enhancer regions

File Name: Supplementary Data 7
Description: Characterization of functional p53 binding sites

File Name: Supplementary Data 8
Description: Pre-processing statistics of PCHi-C datasets.

File Name: Supplementary Data 9
Description: Distal target genes

File Name: Supplementary Data 10
Description: Gene set enrichment analysis

File Name: Supplementary Data 11
Description: Primers
