# Supplementary material for: p53 rapidly restructures 3D chromatin organization to trigger a transcriptional response
Source: Nat Commun. 2024 Apr 1;15:2821. doi: 10.1038/s41467-024-46666-1 (PMC10984980; doi:10.1038/s41467-024-46666-1)
Supplement: Supplementary file 15 — Reporting Summary [file 41467_2024_46666_MOESM15_ESM.pdf]

nature portfolio

Corresponding author(s): Biola M. Javierre

Last updated by author(s): 2023/12/25

Reporting Summary

Nature Portfolio wishes to improve the reproducibility of the work that we publish. This form provides structure for consistency and transparency in reporting. For further information on Nature Portfolio policies, see our Editorial Policies and the Editorial Policy Checklist.

Statistics

For all statistical analyses, confirm that the following items are present in the figure legend, table legend, main text, or Methods section.

n/a	Confirmed	
☐	☒	The exact sample size (*n*) for each experimental group/condition, given as a discrete number and unit of measurement
☐	☒	A statement on whether measurements were taken from distinct samples or whether the same sample was measured repeatedly
☐	☒	The statistical test(s) used AND whether they are one- or two-sided<br>*Only common tests should be described solely by name; describe more complex techniques in the Methods section.*
☒	☐	A description of all covariates tested
☐	☒	A description of any assumptions or corrections, such as tests of normality and adjustment for multiple comparisons
☐	☒	A full description of the statistical parameters including central tendency (e.g. means) or other basic estimates (e.g. regression coefficient) AND variation (e.g. standard deviation) or associated estimates of uncertainty (e.g. confidence intervals)
☐	☒	For null hypothesis testing, the test statistic (e.g. *F*, *t*, *r*) with confidence intervals, effect sizes, degrees of freedom and *P* value noted<br>*Give P values as exact values whenever suitable.*
☒	☐	For Bayesian analysis, information on the choice of priors and Markov chain Monte Carlo settings
☒	☐	For hierarchical and complex designs, identification of the appropriate level for tests and full reporting of outcomes
☒	☐	Estimates of effect sizes (e.g. Cohen's *d*, Pearson's *r*), indicating how they were calculated

Our web collection on statistics for biologists contains articles on many of the points above.

Software and code

Policy information about availability of computer code

Data collection	No software was used for data collection.
Data analysis	Software versions:HiCUP 0.8.2; CHiCAGO 1.14.0; AutoClass 3.3.6; bowtie2 2.3.2; ; WashU Epigenome Browser Legacy 46.2; liftOver tool (Nov 2021 release); 'CRISPR Guide RNA Design Tool' (https://www.benchling.com); Watson Pragmatic algorithm platform provided by FlowJo v1086; Image Studio Lite v5.2; QuantStudio software (v.1.3); TADbit pipeline (specific version: https://github.com/fransua/TADbit/tree/p53_javierre); GEM3 mapper; samtools (1.9); deepTools (3.2.1); Macs2 (2.2.7.1); csaw (1.30.1); DESeq2 (1.36.0); Trim Galore (0.6.6); GenomicRanges (1.50.2); clusterProfiler (4.4.4); msigdbr R package (7.5.1); SciPy v1.10.1; ggplot2 and matploltib v3.7.1; stats (4.2.1).All custom code and the required software versions are publicly available in GitHub at https://github.com/JavierreLab/p53.

For manuscripts utilizing custom algorithms or software that are central to the research but not yet described in published literature, software must be made available to editors and reviewers. We strongly encourage code deposition in a community repository (e.g. GitHub). See the Nature Portfolio guidelines for submitting code & software for further information.

Data

Policy information about availability of data

All manuscripts must include a data availability statement. This statement should provide the following information, where applicable:

- Accession codes, unique identifiers, or web links for publicly available datasets
- A description of any restrictions on data availability
- For clinical datasets or third party data, please ensure that the statement adheres to our policy

Raw and processed data sequencing data for Hi-C, ChIP-seq, RNA-seq and PCHi-C have been deposited in Gene Expression Omnibus (GEO) under the accession number GSE235947.

Research involving human participants, their data, or biological material

Policy information about studies with human participants or human data. See also policy information about sex, gender (identity/presentation), and sexual orientation and race, ethnicity and racism.

Reporting on sex and gender	Our study and findings do not take into consideration sex or gender, nor our findings apply to only one sex or gender due to the nature of the samples and the analysis.
Reporting on race, ethnicity, or other socially relevant groupings	Our study and findings do not take into consideration race, ethnicity, or other socially relevant groupings
Population characteristics	n/a
Recruitment	No participants / volunteers were recruited for our research
Ethics oversight	n/a

Note that full information on the approval of the study protocol must also be provided in the manuscript.

Field-specific reporting

Please select the one below that is the best fit for your research. If you are not sure, read the appropriate sections before making your selection.

☒ Life sciences ☐ Behavioural & social sciences ☐ Ecological, evolutionary & environmental sciences

For a reference copy of the document with all sections, see nature.com/documents/nr-reporting-summary-flat.pdf

Life sciences study design

All studies must disclose on these points even when the disclosure is negative.

Sample size	As sample size we used 2 biological replicates for each condition. Sample size was determined based on similar omics experiments in previous studies, allowing a reduction of heterogeneity bias while keeping the experiments at a non-prohibitive cost.
Data exclusions	No data was excluded from the analysis
Replication	3 relicates were performed. Experimental reproducibility between libraries was measured using reproducibility-score.
Randomization	Randomization is not relevant as we did not allocate datasets into experimental groups
Blinding	Blinding is not necessary as we did not allocate datasets into experimental groups

Reporting for specific materials, systems and methods

We require information from authors about some types of materials, experimental systems and methods used in many studies. Here, indicate whether each material, system or method listed is relevant to your study. If you are not sure if a list item applies to your research, read the appropriate section before selecting a response.

Materials & experimental systems

n/a	Involved in the study	
☐	☒	Antibodies
☐	☒	Eukaryotic cell lines
☒	☐	Palaeontology and archaeology
☒	☐	Animals and other organisms
☒	☐	Clinical data
☒	☐	Dual use research of concern
☒	☐	Plants

Methods

n/a	Involved in the study	
☐	☒	ChIP-seq
☒	☐	Flow cytometry
☒	☐	MRI-based neuroimaging

Antibodies

Antibodies used

WB antobodies: p53 DO1 (Santa Cruz Biotechnology, sc-126, diluted 1:200), p53(phospho S46) (Abcam, ab76242, diluted 1:5000), CDKN1A (Abcam, ab109520, diluted 1:1000), RAD21 (Abcam, ab992, diluted 1:1000), Vinculin (Abcam, ab129002, diluted 1:10000), H3 (Thermo Fisher Scientific, PA5-16183, diluted 1:4000), α-Tubulin (SIGMA-ALDRICH, T6199, diluted 1:10000). The following secondary antibodies were used: IRDye 800CW Goat anti-rabbit IgG secondary antibody (LICOR #926-32211, dilute 1:10000) and IRDye 680RD Goat anti-mouse IgG secondary antibody (LICOR #926-68070, dilute 1:10000).

ChIP-seq antobodies: (1ug of α-H3K27ac and 0.5ug of α-H3K4me1; Diagenode #C15410196 and #C15410194 respectively).

Validation

All primary antibodies were shown to react to their target protein on the manufacturer's website.

Eukaryotic cell lines

Policy information about cell lines and Sex and Gender in Research

Cell line source(s)

HCT116 (RRID:CVCL_0291), HCT116-RAD21-mAC, SH-SY5Y (RRID: CVCL_0019), HepG2 (RRID:CVCL_0027), MCF-7 (RRID:CVCL_0031), C32 (RRID:CVCL_1097), CAKI-1 (RRID:CVCL_0234), Human Umbilical Vein Endothelial Cells (HUVEC; Lonza cat: C2519A), Human Dermal Lymphatic Endothelial Cells (HDLEC) adult (Promocell cat: C-12217), Human Pericytes from Placenta (hPC-PL; Promocell, cat: C-12980)

Authentication

Non of the cells lines were authenticated

Mycoplasma contamination

Cell lines tested negative for mycoplasma

Commonly misidentified lines
(See ICLAC register)

n/a

Plants

Seed stocks

n/a

Novel plant genotypes

n/a

Authentication

n/a

ChIP-seq

Data deposition

☒ Confirm that both raw and final processed data have been deposited in a public database such as GEO.

☒ Confirm that you have deposited or provided access to graph files (e.g. BED files) for the called peaks.

Data access links
May remain private before publication.

Gene Expression Omnibus (GEO) under the accession number GSE235947.

Files in database submission

Hi-C: 0h, 1h, 4h, 7h, 10h, 24h, wash
RNAseq: 0h, 1h, 10h

ChIP-seq (h3K27ac, H3K4me1 and IgG): 0h, 1h, 10h
PCHi-C: 0h, 1h, 10h, RAD21 KD

Genome browser session (e.g. UCSC)

n/a

Methodology

Replicates	3 relicates were performed. Experimental reproducibility between libraries was measured using reproducibility-score.
Sequencing depth	Following ENCODE guide
Antibodies	ChIP-seq antobodies: (1ug of α-H3K27ac and 0.5ug of α-H3K4me1; Diagenode #C15410196 and #C15410194 respectively).
Peak calling parameters	Macs2 (2.2.7.1) was used for peak calling in the narrow mode for H3K27ac and broad mode for H3K4me1, using an input sample as control, with default parameters. Consensus peaks were computed for each condition using Macs2 with all replicates and their respective input samples as control, setting the parameter --scale-to small.
Data quality	Low-quality reads, reads overlapping the ENCODE blacklist, and duplicate reads were filtered out using samtools (1.9). Genome-wide coverage was computed using the function bamCoverage from deepTools (3.2.1). Library size factors for normalization were calculated based on the background signal. This background signal was quantified per sample, excluding the set of previously defined non-redundant enriched regions. Specifically, this signal was quantified on fragment counts over genomic bins of 10kb using the function windowCounts from R package csaw96. Library statistics were assessed using FastQC and MultiQC98 and summarized in Suppl. Data 4
Software	Trim Galore (0.6.6), bowtie2 (2.3.2) in the --very-sensitive mode, samtools (1.9), deepTools (3.2.1), Macs2 (2.2.7.1), csaw (1.30.1), DESeq2 (1.36.0)
